# Space GlucoseControl with the incorporated enhanced model predictive control algorithm is a safe and reliable tool for glycaemic control in medical ICU patients

**DOI:** 10.1186/cc9821

**Published:** 2011-03-11

**Authors:** K Amrein, M Ellmerer, R Hovorka, KH Smolle, N Kachel, TR Pieber, J Plank

**Affiliations:** 1 Medical University of Graz, Austria; 2 BBraun, Melsungen, Germany; 3 University of Cambridge, Institute of Metabolic Science, Cambridge, UK; 4 Joanneum Research, Graz, Austria

## Introduction

Glycaemic control remains an important therapeutic goal in critically ill patients; however, safety and workload are important concerns in its implementation. The enhanced model predictive control (eMPC) algorithm has demonstrated efficacy and safety in critically ill medical and surgical patients. It is integrated in the BBraun Space GlucoseControl system (SGC, project title: Space TGC) which consists of three Space pumps (two for nutrition, one for insulin). A central user interface (Space Control) and central hardware connected to Space Control (SGC Module) provide suggestions for insulin rate and glucose measurement interval.

## Methods

Performance of SGC was tested in mechanically ventilated medical ICU patients for up to 14 days. It was operated by 54 trained nurses and the target range was 80 to 150 mg/dl (4.4 to 8.3 mmol/l). Patients with an expected ICU stay >3 days were recruited in this single-centre, noncontrolled trial.

## Results

From February to November 2010, 18 patients (age 63 ± 17, BMI 29.1 ± 7.3, APACHE II 26 ± 7, 13 male, four diabetic) were included for a period of 7.0 ± 3.7 days and 1,583 blood glucose values were analysed, corresponding to a sampling interval of 2 hours. The percentage of glucose values within predefined ranges was as follows: ≤40 mg/dl: 0.0%; >40 and <60 mg/dl: 0.3%; ≥60 and <80 mg/dl: 4.3%; ≥80 and ≤150 mg/dl: 74.7%; and >150 mg/dl: 20.7%. Mean arterial blood glucose was 127 ± 35 mg/dl (7.0 ± 2.0 mmol/l). No hypoglycaemic episodes (≤40 mg/dl) occurred during the trial.

## Conclusions

Performance of SGC with incorporated eMPC algorithm was excellent. Seventy-five per cent of all glucose values were within the target range and no hypoglycaemic episodes occurred. SGC is a safe and reliable method to control blood glucose in critically ill patients in the medical ICU.

